# Cerebellum Susceptibility to Neonatal Asphyxia: Possible Protective Effects of N-Acetylcysteine Amide

**DOI:** 10.1155/2018/5046372

**Published:** 2018-01-30

**Authors:** T. Benterud, S. Manueldas, S. Rivera, E. Henckel, E. M. Løberg, S. Norgren, L. O. Baumbusch, R. Solberg, O. D. Saugstad

**Affiliations:** ^1^Department of Pediatric Research, Division of Pediatric and Adolescent Medicine, Oslo University Hospital, Rikshospitalet, Oslo, Norway; ^2^Department for Surgical Research, Oslo University Hospital, Rikshospitalet, Oslo, Norway; ^3^University of Oslo, Oslo, Norway; ^4^Aix Marseille Université, CNRS, NICN, Marseille, France; ^5^Department of Clinical Science, Intervention and Technology, Karolinska Institutet, Stockholm, Sweden; ^6^Department of Neonatology, Karolinska University Hospital, Stockholm, Sweden; ^7^Department of Pathology, Oslo University Hospital, Ullevål, University of Oslo, Oslo, Norway; ^8^Department of Women's and Children's Health, Division of Paediatric Endocrinology, Karolinska Institutet, Stockholm, Sweden; ^9^Department of Pediatrics, Vestfold Hospital Trust, Tønsberg, Norway

## Abstract

**Background:**

After perinatal asphyxia, the cerebellum presents more damage than previously suggested.

**Objectives:**

To explore if the antioxidant N-acetylcysteine amide (NACA) could reduce cerebellar injury after hypoxia-reoxygenation in a neonatal pig model.

**Methods:**

Twenty-four newborn pigs in two intervention groups were exposed to 8% oxygen and hypercapnia, until base excess fell to −20 mmol/l or the mean arterial blood pressure declined to <20 mmHg. After hypoxia, they received either NACA (NACA group, *n* = 12) or saline (vehicle-treated group, *n* = 12). One sham-operated group (*n* = 5) served as a control and was not subjected to hypoxia. Observation time after the end of hypoxia was 9.5 hours.

**Results:**

The intranuclear proteolytic activity in Purkinje cells of asphyxiated vehicle-treated pigs was significantly higher than that in sham controls (*p* = 0.03). Treatment with NACA was associated with a trend to decreased intranuclear proteolytic activity (*p* = 0.08), There were significantly less mutations in the mtDNA of the NACA group compared with the vehicle-treated group, 2.0 × 10^−4^ (±2.0 × 10^−4^) versus 4.8 × 10^−5^(±3.6 × 10^−4^, *p* < 0.05).

**Conclusion:**

We found a trend to lower proteolytic activity in the core of Purkinje cells and significantly reduced mutation rate of mtDNA in the NACA group, which may indicate a positive effect of NACA after neonatal hypoxia. Measuring the proteolytic activity in the nucleus of Purkinje cells could be used to assess the effect of different neuroprotective substances after perinatal asphyxia.

## 1. Introduction

Globally, approximately 45% of the cases of child death within the first five years of life occur during the neonatal period [1]. Despite the numbers of fatal cases due to the complications of perinatal asphyxia have been remarkably reduced over the last 15 years, there are still many children suffering from extensive neurological consequences after perinatal asphyxia.

It is widely recognized that in neonatal basal ganglia, the cerebral cortex, thalamus, and hippocampus are the most vulnerable brain areas after perinatal hypoxia [2]. However, improved neuroimaging modalities have shown that the cerebellum is more damaged after perinatal asphyxia than previously suggested [3]. In addition to being a coordinator of motor function, the cerebellum plays a role in higher cognitive functions and several authors argue that the abnormalities in the cerebellum may play a pivotal role in different mental disorders, such as attention deficit and hyperactivity disorder (ADHD) and schizophrenia [4, 5].

Our group has recently described anti-inflammatory and possible neuroprotective effects of the antioxidant N-acetylcysteine amide (NACA) after neonatal hypoxia-reoxygenation in a neonatal pig model. Further, NACA reduced the levels of the proinflammatory cytokine IL-1*β* and the transcription factor NF-*κ*B in the prefrontal cortex of the brain after neonatal hypoxia-reoxygenation [6]. The substance NACA has many similarities with N-acetylcysteine, which has been used as an antioxidant precursor to glutathione in the treatment of paracetamol overdose for more than 30 years [7]. However, due to the amide group which increases its lipophilicity, NACA has an augmented ability to penetrate the blood-brain barrier and the cellular membranes [8, 9]. Moreover, we showed in a pig epithelial-like embryonic EFN-R kidney cell line that NACA had a protective effect on cells exposed to H_2_O_2_-induced oxidative stress [10].

In the present study, we wanted to explore if NACA treatment after hypoxia-reoxygenation reduces cerebellar injury, using the same group of pigs. The model is well established, and it has been used for years in our department to induce oxidative stress [11].

Matrix metalloproteinases (MMPs) display proinflammatory activity and exert deleterious actions in numerous neuropathological settings, including hypoxia and ischemia [12, 13]. Moreover, some MMPs have been located in the nucleus of neural cells [14, 15] and are associated with neuronal DNA degradation upon oxygen-glucose deprivation [16]. Therefore, we used in situ zymography, which reflects the net metalloproteinase activity in the tissue, to measure proteolytic activity in the cerebellum upon hypoxia-reoxygenation.

Furthermore, reactive oxygen species (ROS) produced during and after perinatal asphyxia may induce lesions of mitochondrial DNA (mtDNA) and subsequently lead to impaired function of neural cells [17]. In this study, we investigated mtDNA in the cerebellum after hypoxia-reoxygenation and if the pigs subjected to NACA after hypoxia (NACA group) would be less susceptible to mutations of mtDNA. The objective of the present study was to evaluate the damage-reduction potential of NACA on cerebellar injury after hypoxia-reoxygenation in neonatal pigs.

## 2. Methods

### 2.1. Study Design

**Figure 1:**
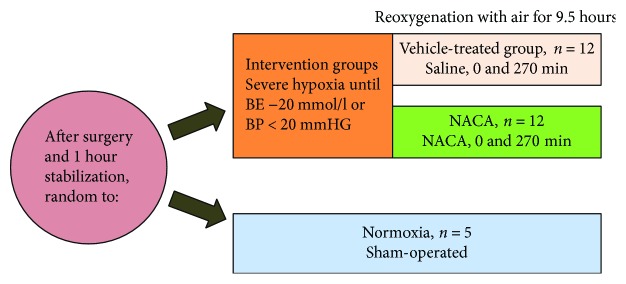
Experimental protocol: twenty-nine pigs were included, twelve in each intervention group and five in the sham group. BE = base excess and BP = blood pressure.

A total of 29 newborn pigs, age 12–36 hours, hemoglobin >5 g/dl, and in good general condition, were included in this study (Figure 1). The pigs were anesthetized, ventilated, and surgically prepared, including insertions of central venous and arterial lines, as previously described by Benterud et al. [18].

The experimental protocol has been thoroughly described in our previous article [6]. Briefly summarized, twenty-four pigs were randomized into two intervention groups. Both groups were subjected to 8% oxygen until base excess (BE) values declined to −20 mmol/l or mean arterial blood pressure (MABP) fell below 20 mmHg. During hypoxia, CO_2_ was added, to achieve a PaCO_2_ of 8.0–9.5 kPa, in order to imitate perinatal asphyxia. At the end of hypoxia, 12 of the pigs were treated with NACA 300 mg/kg diluted in saline 0.9%, while the other 12 received normal saline (vehicle-treated group). An additional dose of NACA or saline was administered 270 minutes after the hypoxic challenge. The pigs were reoxygenated with air for 9.5 hours until they were terminated with an overdose of pentobarbital 150 mg/kg.

The five pigs in the sham-operated group underwent the same procedures as described in our previous article and were not exposed to hypoxia.

Due to the fact that there might be some subtle gender differences between neonatal pigs [19], the same number of male and female animals were included in each group.

## 3. Laboratory Methods

### 3.1. In Situ Zymography

We focused our investigations of the cerebellum on Purkinje cells, due to their high vulnerability to hypoxia [20, 21]. Furthermore, the nucleus of Purkinje cells presented the highest level of fluorescence and seemed to be particularly affected by hypoxia-reoxygenation. In situ zymography is commonly used as an index of net metalloproteinase activity resulting from the balance between gelatinases (principally MMP-9 and MMP-2) and the tissue inhibitors of MMPs (TIMPs) that are present in the tissue. In situ zymography was performed to localize net gelatinolytic activity in cerebellar brain sections, with minor modifications compared to the method previously described for brain tissue [22]. Sections of fresh frozen brain tissue (20 *μ*m thick) from the cerebellum were generated using a cryostat (Leica CM3050S, Nussloch, Germany). Nonfixed brain sections were incubated for 2 hours at 37°C in a humid dark chamber in a reaction buffer that contained 0.5 M Tris-HCl, 1.5 M NaCl, 50 mM CaCl_2_, 2 mM sodium azide (pH 7.6), and 80 *μ*g/ml of intramolecularly quenched FITC-labeled DQ-gelatin (EnzCheck collagenase assay kit; Thermo Fisher Scientific, Waltham, Massachusetts, USA). After the incubation, the tissue was fixed in 4% paraformaldehyde Antigenfix solution (Diapath, MM France, Brignais, France), incubated for 5 minutes with 0.5 *μ*g/ml Hoechst 33258 (Thermo Fisher Scientific), and mounted in Prolong Gold Antifading reagent (Thermo Fisher Scientific). The sections were incubated with 1 mM phenanthroline (Thermo Fisher Scientific), a broad-spectrum metalloproteinase inhibitor. Samples were observed with a confocal microscope (LSM 700 Zeiss, Jena, Germany), and images were analyzed using the Zen (Zeiss) and ImageJ softwares (NIH, Bethesda, MD, USA). Gelatin-FITC cleavage by tissue gelatinases releases quenched fluorescence representative of net proteolytic activity. Sections incubated without DQ-gelatin were not fluorescent. We used 8 piglets per experimental group and 5 from the control group, and we analyzed three slices per animal. The 8 pigs in each experimental group were randomly selected.

### 3.2. Histopathology

After removal of the brain, one hemisphere was immersion fixed in formalin. Tissue blocks (0.5 cm thick) from the cerebellum were embedded in paraffin, sliced in 4 *μ*m thick sections, and stained with hematoxylin and eosin (H&E). An experienced neuropathologist evaluated the slices. Because of suboptimal conservation of the cerebellum, a simplified assessment was conducted. Cerebellar damage was categorized into two variables: (1) generalized damage and (2) localized/no damage.

The term generalized was used if the injuries were observed in all parts of the tissue section, in comparison to localized, where only small and limited parts of the tissue section were involved. Due to the limited amount of tissue, 23 pigs were evaluated, 5 in the sham group and 18 in the intervention groups. Of the pigs in the intervention groups, 8 were in the NACA group and 10 in the vehicle-treated group.

### 3.3. DNA Extraction from Cerebellum

Total DNA from the cerebellum was isolated using DNA blood and tissue kit (Qiagen, Hildesheim, Germany). 10–25 mg of tissue from each pig was lysed and dissolved according to manufacturer's protocol with slight modifications (For a more detailed description, please read the Supplementary Materials (available here)).

### 3.4. Mutation Rate of Mitochondrial DNA

Random mutation capture (RMC) was performed to assess the rate of mutations of mtDNA in the cerebellum. The method is thoroughly described in the Supplementary Materials section.

### 3.5. Gene Expression

Real-time quantitative PCR (RT-qPCR) was performed to investigate the expression levels of genes involved in the NLRP3 inflammatory pathway, including IL-1*β*, IL18, and NLRP3. RT-qPCRs were performed using the RT-RNA PCR kit following the instruction of the producer (Applied Biosystems, now Life 21 Technologies, Carlsbad, CA, USA). The final reaction volume was 25 *μ*l, including 12.5 *μ*l of universal master mix (Life 21 Technologies), 200 nmol of forward and reverse primers, and 5 *μ*l of the diluted cDNA product (1:12.5 dilutions). The 96-well plate reactions were carried out with an initial cycle at 50°C for 2 minutes, a heating stop at 95°C for 10 minutes, followed by 45 cycles of 30 seconds at 95°C, and 60 seconds at 60°C. All reactions were performed on a Vii7 Sequence Detection System (Life 21 Technologies). Experiments were performed in triplets, and all transcript quantification data were normalized to the endogenous reference gene P0.

The primer sequences 5′–3′ were as follows: NLRP3 (forward primer) AAAAGCCTGAGTTGACCATTGTC and (reverse primer) CACTATCACTTATACACACCCAGATGTC; IL-1*β* (forward primer) GTGATGCCAACGTGCAGTCT and (reverse primer) GTGGGCCAGCCAGCACTA; and IL-18 (forward primer) GCCTCACTAGAGGTCTGGCAGTA and (reverse primer) GGACTCATTTCCTTAAAGGAAAGAGTT.

### 3.6. ELISA

To determine the protein concentrations of IL-1*β*, enzyme immunoassays kit was used as instructed by the manufacturer (R&D Systems, Oxford, UK).

## 4. Statistical Analysis

The analyses were performed using SPSS software v21 (SPSS Inc., Chicago, IL, USA). The data were analyzed using the Kruskal-Wallis test, Mann–Whitney *U* test, or Student *t*-test with winsorizing for variables with nonnormal distributions, ANOVA, and an independent sample *t*-test for normal distributions. Levene's test for equality of variance was performed before the *t*-test. If Levene's test documented a significant variance difference between the compared groups, a *t*-test assuming different variances was performed. Otherwise, a *t*-test assuming equal variance was performed.

All the differences were considered significant if *p* < 0.05. When calculating the results of the histopathological evaluation, chi-square test without Yates' correction was performed.

## 5. Results

We did not find any significant difference between the genders, and therefore, the data for both genders are merged.

### 5.1. Physiological Parameters

At baseline, there were no differences in weight, hemoglobin, pH, BE, lactate, pCO_2_, or glucose level between the groups. Arterial blood gases were taken at 6 different time points during the experiment. There were no significant differences between the 2 intervention groups in any of these variables. The physiological parameters and their change during the experiments are thoroughly described in Table 1.

### 5.2. In Situ Zymography

**Figure 2:**
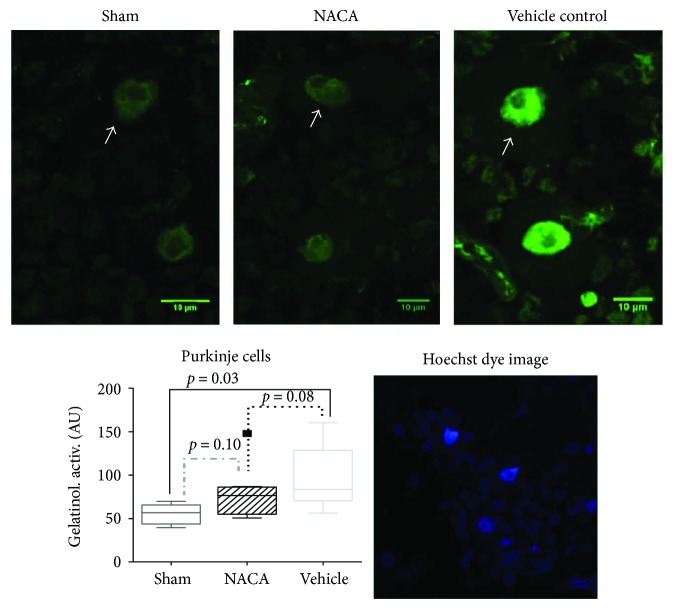
In situ zymography of the cerebellum. Net in situ gelatinolytic activity increases in the nucleus of Purkinje cells in the cerebellum after hypoxia-resuscitation. Fluorescence photomicrographs of cerebellar sections displaying in situ zymography in pigs who were sham-operated, exposed to NACA after hypoxia, and vehicle-treated groups. Intranuclear fluorescence signal in Purkinje cells (white arrow) represents the proteolytic activity (green). An increase in fluorescence signal strength represents a higher degree of proteolytic activity. The graph represents the quantification of net gelatinolytic activity (in arbitrary units (AU) of fluorescence) for sham 52 (±12), NACA group 72 (±16), and vehicle-treated group 97 (±36). Values are given as mean ± SD. Hoechst dye was used as a nuclear marker (blue). Images are representative of pictures obtained from each group.

Compared to sham animals, a significant increased proteolytic activity was found in animals exposed to hypoxia alone (vehicle group, *p* = 0.03), by contrast to NACA animals where a significant difference was not found (*p* = 0.08). Representative images obtained from five (sham) and eight (NACA and vehicle-treated groups) animals in each group are shown in Figure 2.

### 5.3. Histopathology

Significantly, more pigs in the intervention groups had generalized damage than in the control group (*p* < 0.05) (Table 2).

There was no difference between the two intervention groups (*p* = 0.67).

**Figure 3:**
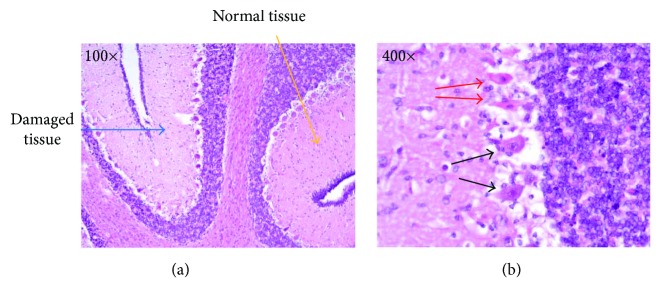
An example of localized damage in the cerebellum from one pig (two different magnifications 100x and 400x). In (b), the red arrows point to eosinophilic Purkinje cells, representing neurons with hypoxic injury. The black arrows point to normal Purkinje cells.

Figure 3 shows an example of the cerebellum of a pig with a localized damage in different magnifications.

### 5.4. mtDNA Mutation

**Figure 4:**
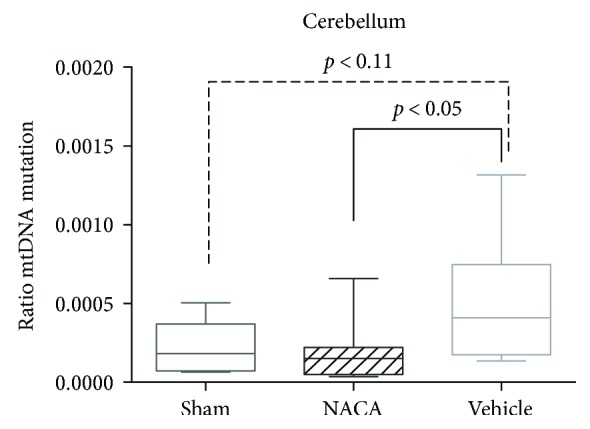
The picture depicts mtDNA mutations in the cerebellum. Values are given as mean ± SD. When comparing the ratios of mutations between the different groups, there was a significantly lower ratio of mutations in pigs exposed to NACA after severe hypoxia than in vehicle-treated group (2.0 × 10^−4^, SD ± 2.0 × 10^−4^ versus 4.9 × 10^−4^, SD ± 3.6 × 10^−4^), *p* < 0.05.

There were significantly fewer mutations in the NACA group than in the vehicle-treated group (2.0 × 10^−4^, SD ± 2.0 × 10^−4^ versus 4.9 × 10^−4^, SD ± 3.6 × 10^−4^) (*p* < 0.05) (Figure 4).

There was no significant difference when comparing the mtDNA mutation rate between the sham (2.2 × 10^−4^, SD ± 1.7 × 10^−4^) and vehicle-treated groups (*p* = 0.11); however, the sham group consists of only five pigs.

### 5.5. Quantitative Real-Time PCR (qRT-PCR)

Between the groups, there were no significant differences in gene expression of NLRP3, IL-18, and IL-1*β*.

### 5.6. Protein Concentrations of IL-1*β* in the Cerebellum

The concentrations of IL-1*β* did not differ between the groups. In the NACA group, the concentration was 15.9 ± 9.6 versus 16.9 ± 8.2 in the vehicle-treated group (*p* = 0.82). A figure of the concentrations of IL-1*β* is included in the Supplementary Materials.

## 6. Discussion

To our knowledge, the present study is the first to use in situ zymography of the cores of Purkinje cells as a marker of hypoxic damage. Measuring the net gelatinolytic activity may be a relevant method to assess the grade of inflammation in cerebellar tissue. Net gelatinolytic activity reflects the proteolytic activity of gelatinases in a specific tissue [22]. The proteolytic activity of MMPs is tightly associated with the activity of TIMPs. Altered balance between MMPs and TIMPs results in a less-controlled equilibrium and may lead to an abrupt increase of proteolysis and pathological processes, including inflammation [12, 13]. This assumption has been strengthened by numerous observations relating to increases in gelatinase activity with glial reactivity and neuronal demise and has been demonstrated in rodents after global cerebral ischemia [22, 23] and excitotoxic seizures induced by kainate [24]. In the latter model, gelatinolysis increased in neurons as early as eight hours after excitotoxic insult and remained high for several days in blood vessels and reactive glial cells of vulnerable areas, in relation with neuroinflammation.

Moreover, Chen et al. showed that exposing newborn rats to a broad-spectrum inhibitor of MMPs after hypoxia-ischemia provided a long-term protection in both neuronal morphology and neurological function in the immature brain [25]. Furthermore, Zhang et al. recently showed that net gelatinolytic activity was significantly increased after inflicted traumatic brain injury in a rodent model [26]. Taking these findings into consideration, we suggest that measuring net gelatinolytic activity in the nucleus of Purkinje cells has a stronger association with damage of the neurons than measuring mRNA or protein levels for gelatinases, because gelatinolysis provides the balance between proteolysis and its inhibition. Further on, we did not perform subcellular biochemical fractionation, since that could have compromised other analyses, for instance IL-1*β*.

Our group has previously investigated the association between net gelatinolytic activity and gene expression of MMP-2 and MMP-9 in various tissues, such as the liver, lungs, and striatum [27, 28]. The gene expression of MMP-2 and MMP-9, as well as the protein levels of active MMP-2 and MMP-9, was associated with increased activity in the liver and lungs, whereas no such association was found in the striatum. Due to the important role of Purkinje cells in the developing brain [29], we speculate that the analysis of gelatinolytic activity in these cells could serve as an important tool in evaluating the various effects of neuroprotective substances after hypoxia in different models. The observation that pigs treated with NACA had a tendency to lower levels of gelatinolytic activity, compared with the vehicle-treated group may indicate that NACA reduces inflammation in the cerebellum.

Our results are in line with previous investigations of our group by Solberg et al. on the striatum of neonatal piglets, which observed an increased gelatinolytic activity in the nuclear compartment as well as in the cytoplasm of the neurons 9.5 hours after hypoxia. At that time point, the differences between the groups were not visible on HE stainings [30]. Furthermore, Hill detected an increased intranuclear gelatinolytic activity immediately after reoxygenation in a primary culture of cortical neurons after oxygen and glucose deprivation [16]. In the same study, they treated rats with an inhibitor of MMPs before they were subjected to occlusion of the middle cerebral artery. The rats exposed to the MMP inhibitor displayed significantly less apoptosis than the control group. These observations may indicate that the increased intranuclear gelatinolytic activity in neurons, such as Purkinje cells, could be an early marker of future neuronal degeneration.

Regarding the gene expression of NLRP3, IL-1*β*, and IL18, there were no significant changes between the groups. These results could be in line with previous findings by our group exhibiting that for rats exposed to hypoxia and sham-operated rats, the mRNA expression of these components were similar in some cerebral subregions, including the cortex and the subventricular zone, at 24 hours after hypoxia [31]. Therefore, it is not surprising that no variability between the groups was revealed at one specific time point in our study. Further studies should be conducted on the time dependency of the compounds of the NLRP3 inflammasome pathway.

In addition, investigations did not reveal any significant changes between the groups in the levels of IL-1*β* as early as 9.5 hours after hypoxia, which stands in contrast with another report, showing that cerebellar IL-1*β* concentrations were significantly changed for all time points between 3 hours and 7 days in neonatal rats subjected to hypoxia [32]. The differences between these studies could be due to different methodology or simply reflect specific reactions to hypoxic injury across animal species.

An increased production of ROS may cause mutations in the mtDNA leading to a critical effect on the activity of the mitochondrial electron transport chain, which subsequently may lead to mitochondrial dysfunction, apoptosis/necrosis, and diseases [33]. Wang et al. showed that damage to mtDNA may lead to diminished mitochondrial bioenergetics and hamper the maturation of neural stem cells [34]. We speculate that our results consisting of a significant reduced mutation rate of mtDNA in pigs subjected to NACA after hypoxia may be associated with a better neurological outcome, which stands in line with the findings of Patel et al. who demonstrated that NACA preserved mitochondrial bioenergetics and improved functional recovery after inflicted spinal trauma [35]. The lack of significant difference between the sham-operated and vehicle-treated groups could be due to the limited number of pigs included in the sham group (*n* = 5).

Our findings suggest that NACA could reduce the mitochondrial damage and thereby have a positive influence on the energy metabolism of neural cells.

Histopathological analyses of the cerebellum revealed no significant differences between the intervention groups but a significant difference between the sham and the intervention groups. The limited signs of cell death observed in some of the sham pigs may be due to possible harmful effects of anesthesia on the brains of newborns, as described in other publications [36–38]. Moreover, all pigs underwent surgical procedures which could be potentially harmful. On the other hand, some anesthetics may have neuroprotective features and a recent study by Liu et al. demonstrated that midazolam may protect against neuroapoptosis induced by physiological and oxidative stress [39]. The anesthetic regime was similar for each pig, and therefore, these effects should be consistent across all animals.

After severe perinatal hypoxia, a certain degree of cell death will occur, during and immediately after the hypoxic challenge. Between 6 and 24 hours later, a phase of secondary energy failure may evolve with declines in phosphocreatinine and ATP, impaired mitochondrial function, and further neuronal cell death. Many animals in our study have probably not reached the phase of secondary energy failure, and there are few signs of cell death visible on histological sections 9.5 hours after hypoxia. At this time point, however, the mechanistic measures of injury may differ between animals exposed to NACA and those who were vehicle treated. We suggest that if we had run the trials for an extended period of time, we would have seen a difference in histopathology between the two intervention groups.

## 7. Conclusion

Generally, pigs exposed to NACA after hypoxia revealed a tendency to reduced gelatinolytic activity in cerebellar Purkinje cells, measured with in situ zymography and a significant reduction of the mutation rate of mtDNA in the cerebellum. We therefore speculate that NACA may have neuroprotective capabilities after perinatal asphyxia. Our observations are in line with our previous study using the same group of animals, where we described possible anti-inflammatory effects of NACA in the cortex of neonatal pigs [6]. However, more studies are needed before NACA could be considered useful in a neonatal clinical setting.

Finally, our results indicate that using in situ zymography in the investigation of Purkinje cells could be a valuable biomarker to compare different neuroprotective substances in hypoxia-reoxygenation models.

## 8. Limitations of the Study

The mutations of mtDNA were only measured at one time point. Gel zymography of MMPs was not conducted; however, we assume that evaluating the net gelatinolytic activity reflects the actual proteolytic activity better than measuring the activity of one specific MMP. Although animal experiments have largely contributed to our understanding of human pathophysiology, we should be cautious when translating the results from animals to humans. We postulate that the differences between the groups could have been larger if the animals had been observed over a longer time period. Also, we are aware that the inclusion of both genders in a small study could possibly attenuate observable differences.

Other points of concern are that the number of animals in each group was relatively small and the study time was limited to 9.5 hours, so there was no long-term follow-up. Furthermore, electrophysiological surveillance of the pigs with EEG while anesthetized could have provided us with valuable information; however, this was not performed in this trial.

Prior to this investigation, the interindividual distribution of the proteolytic activity and rate of mutations in mitochondrial DNA following asphyxia were unknown. Thus, a proper power calculation to determine the adequate sample size could not be performed. In retrospect, the small sample size is troublesome and limits the statistical analysis.

## Figures and Tables

**Table 1:** Background and physiological parameters throughout the experiment.

	Control *n* = 5	Hypoxia + NACA *n* = 12	Hypoxia + saline *n* = 12
Weight (g)	1923 (±76)	1874 (±184)	1924 (±124)
Hypoxia time (min)	33 (±12)	40 (±13)	33 (±12)
Hb g/100 ml start	7.7 (±1.8)	8.0 (±1.2)	7.0 (±1.0)
Hb g/100 ml end	5.9 (±1.2)	6.3 (±0.8)	7.1 (±2.7)
Gender (male/female)	3/2	6/6	6/6
pH			
Start	7.44 (±0.04)	7.42 (±0.04)	7.45 (±0.04)
End hypoxia	7.43 (±0.05)	6.87 (±0.08)	6.92 (±0.11)
30 min reox	7.46 (±0.04)	7.18 (±0.09)	7.18 (±0.07)
90 min reox	7.45 (±0.06)	7.45 (±0.04)	7.35 (±0.08)
270 min reox	7.42 (±0.12)	7.45 (±0.04)	7.39 (±0.10)
570 min reox	7.38 (±0.06)	7.42 (±0.04)	7.39 (±0.09)
BE (mmol/l)			
Start	4.3 (±2.9)	−0.5 (±4.9)	−0.3 (±3.6)
End hypoxia	4.1 (±3.6)	−18.9 (±2.2)	−19.0 (±3.9)
30 min reox	4.4 (±1.9)	−13.3 (±4.7)	−15.1 (±3.5)
90 min reox	4.2 (±2.3)	−3.9 (±5.0)	−5.7 (±4.6)
270 min reox	2.9 (±4.5)	−2.2 (±4.4)	−1.4 (±5.0)
570 min reox	0.5 (±3.4)	−6.5 (±6.6)	−3.7 (±5.4)
Lactate (mmol/l)			
Start	2.3 (±1.1)	2.3 (±1.1)	2.8 (±1.0)
End hypoxia	2.4 (±2.1)	14.3 (±2.7)	13.2 (±3.1)
30 min reox	1.8 (±1.1)	10.7 (±3.8)	11.2 (±0.6)
90 min reox	1.5 (±0.6)	5.9 (±2.2)	6.3 (±2.1)
270 min reox	1.3 (±0.4)	1.7 (±0.8)	2.3 (±2.2)
570 min reox	1.7 (±1.0)	1.4 (±1.0)	2.1 (±2.0)
Glucose (mmol/l)			
Start	5.0 (±1.2)	6.4 (±2.1)	6.7 (±2.3)
End hypoxia	5.0 (±1.5)	9.6 (±3.4)	9.1 (±3.7)
30 min reox	4.7 (±0.8)	8.0 (±3.5)	7.5 (±3.6)
90 min reox	4.9 (±0.6)	6.5 (±2.3)	6.7 (±2.3)
pCO_2_ (kPa)			
Start	5.0 (±1.1)	5.2 (±0.9)	5.0 (±1.1)
End hypoxia	5.7 (±0.3)	8.4 (±1.4)	7.7 (±1.1)
30 min reox	5.4 (±0.6)	5.1 (±0.8)	4.4 (±0.6)

**Table 2:** 

	Generalized damage	Localized or no damage	Total
Intervention	9	9	18
Sham	0	5	5
Total	9	14	23

## Supplementary Materials

Supplementary Materials Cerebellum susceptibility to neonatal asphyxia: possible protective effects of N-acetylcysteine amide (NACA).
