# Supplementary material for: Cerebellum Susceptibility to Neonatal Asphyxia: Possible Protective Effects of N-Acetylcysteine Amide
Source: Dis Markers. 2018 Jan 30;2018:5046372. doi: 10.1155/2018/5046372 (PMC5831588; doi:10.1155/2018/5046372)
Supplement: Supplementary Materials — Cerebellum susceptibility to neonatal asphyxia: possible protective effects of N-acetylcysteine amide (NACA). [file 5046372.f1.pdf]

Supplementary Materials: Cerebellum susceptibility to neonatal asphyxia: Possible protective effects of N-Acetylcysteine Amide (NACA)

Extraction of mtDNA from cerebellum

All extractions were performed using the Qiagen kit (DNeasy® Blood and Tissue Kit, Hilden, Germany), following the instruction by the producers. Briefly, l*y*sis of the samples was performed by the addition of 180 µl buffer ATL and 20 µl proteinase K and incubation over night at 56°C. Thereafter, 200 µl 100% ethanol was added, the mixture was transferred into a DNeasy Mini spin column placed in a 2 ml collection tube, and centrifuged at 8000 rpm for 1 minute. The spin column was placed into a new 2 ml collection-tube and processed in two separate wash steps with buffers AW1 and AW2. Finally the DNA was eluted by adding 200 µl buffer AE to the center of the spin column membrane and centrifuged for 1 minute at 8000 rpm. Concentration and purity of DNA was assessed by NanoDrop ND 100 (Life Science, USA) and diluted to a final concentration of 6 ng/µl and 15 ng/µl DNA.

The Random Mutation Capture (RMC) method to estimate mtDNA damage

The RMC method is a technique to estimate the mutation rate of mtDNA in a sample, based on a PCR-reaction combined with restriction enzyme digestion [1]. Basically, primers for a PCR-reaction cover a sequence containing a restriction enzyme target site, e.g. TCGA for *TaqI*. A restriction enzyme digestion of the DNA-template prior to the PCR reaction will cut the wild-type template preventing a PCR product, but a mutation in the target site will lead to a PCR-reaction product. The ratio between the amount of wild-type (WT) (cut) and mutation (uncut) product can be estimated, if the total amount of template is determined by another PCR-reaction product with primers covering a close-by sequence excluding the restriction enzyme cutting site for the respective restriction enzyme (figure 1). The product of a wild-type (WT) and the mutant template will result in different ct-value signals in a qRT-PCR reaction, the mutant (uncutted) product will display a higher ct-value than the WT (cutted). The mutation rate is calculated with $\Delta\Delta^{ct}$ method.

Figure 1

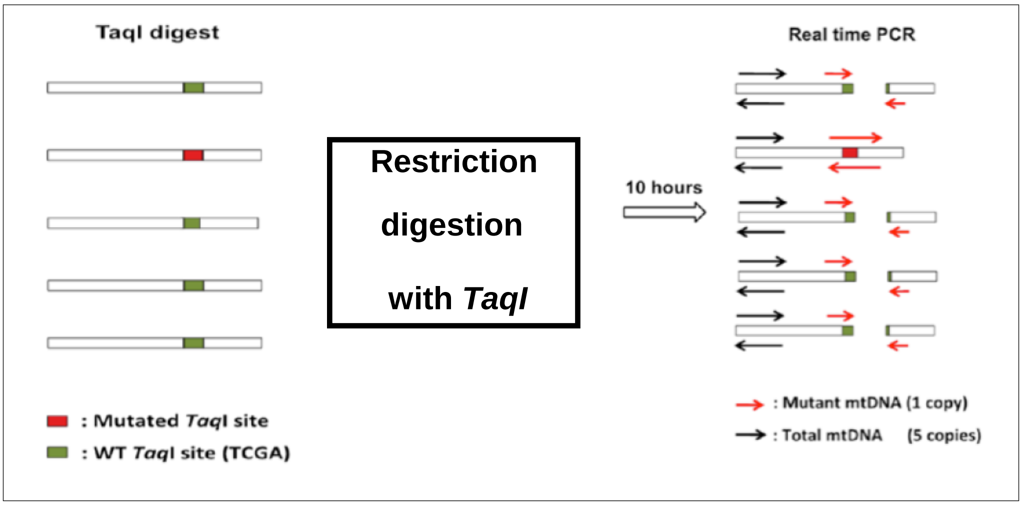

Restriction digestion with *TaqI* enzyme in mitochondrial *12S* gene. *TaqI* restriction enzyme recognizes T^CGA and cuts at this site.

mtDNA Restriction Enzyme test

Primers were designed using the primer 3 plus program and the efficiency tested by a 10-fold qRT-PCR dilution reaction using the pet101 plasmid (Invitrogen, Paisley, UK) and cerebellum samples. The correct fragment size was confirmed and the mtDNA samples were digested using 1U restriction enzyme *TaqI* (Invitrogen, Paisley, UK) with smart cut buffer and nuclease free water in a total volume of 30 µl. The restriction digestion was performed at 65°C for 15 minutes followed by inactivation of the enzyme for 10 minutes at 95°C.

The primer sequences for mtDNA 12S were: Forward primer (5´-3´): CGCAACTGCCTAAACTCAA,

Reverse primer (5´-3´): TAGCCCATTTCTTTCCAACC

Analyses of mtDNA damage

The mastermix for one reaction contained 6x SYBR green, 0.5 µM forward- and reverse primer and nuclease free water to a final volume of 20 µl.To mastermix with target *12S* gene. 1U *TaqI* enzyme was added.

The digestion step was performed at 15 minutes at 65°C for enzyme digest and 10 minutes at 95°C for inactivation.

The product was analyzed with qRT-PCR. DNA mutation frequency was calculated using ΔCT method following, 2exp- (ct_{targ} –ct_{ctrl}), where ct_{Targ} and ct_{ctrl} represent CT values of enzyme-treated and non-treated DNA.

Figure 2

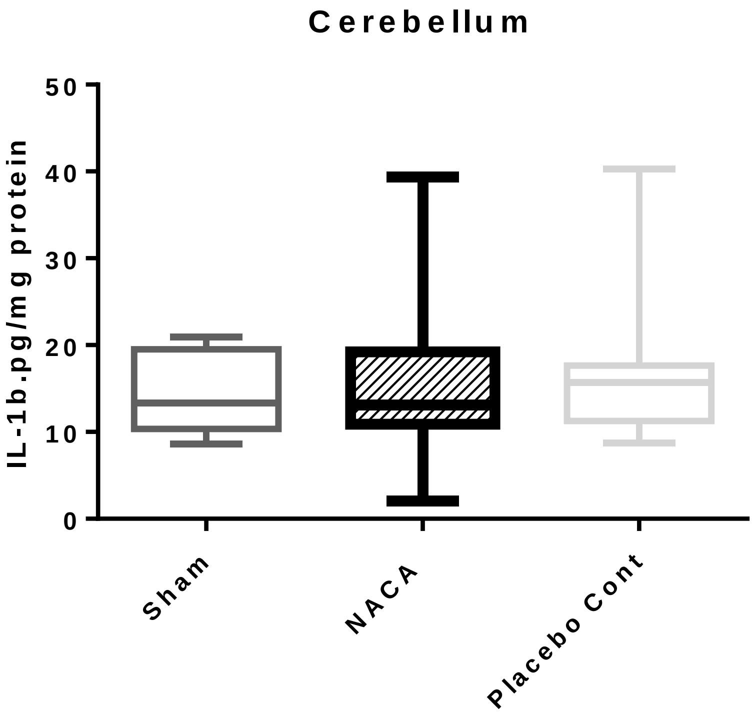

The figure display the concentrations of IL-1β in each group

Reference:

1 Vermulst M, Bielas JH, Loeb LA: Quantification of random mutations in the mitochondrial genome. Methods (San Diego, Calif) 2008;46:263-268.
